# Fabrication and thermo-mechanical behavior of ultra-fine porous copper

**DOI:** 10.1007/s10853-014-8622-4

**Published:** 2014-09-30

**Authors:** Marius Kreuzeder, Manuel-David Abad, Mladen-Mateo Primorac, Peter Hosemann, Verena Maier, Daniel Kiener

**Affiliations:** 1 Department of Materials Physics, Montanuniversität Leoben, 8700 Leoben, Austria; 2 Department of Nuclear Engineering, University of California, Berkeley, CA 94720 USA

## Abstract

Porous materials with ligament sizes in the submicrometer to nanometer regime have a high potential for future applications such as catalysts, actuators, or radiation tolerant materials, which require properties like high strength-to-weight ratio, high surface-to-volume ratio, or large interface density as for radiation tolerance. The objective of this work was to manufacture ultra-fine porous copper, to determine the thermo-mechanical properties, and to elucidate the deformation behavior at room as well as elevated temperatures via nanoindentation. The experimental approach for manufacturing the foam structures used high pressure torsion, subsequent heat treatments, and selective dissolution. Nanoindentation at different temperatures was successfully conducted on the ultra-fine porous copper, showing a room temperature hardness of 220 MPa. During high temperature experiments, oxidation of the copper occurred due to the high surface area. A model, taking into account the mechanical properties of the copper oxides formed during the test, to describe the measured mechanical properties in dependence on the proceeding oxidation was developed. The strain rate sensitivity of the copper foam at room temperature was ∼0.03 and strongly correlated with the strain rate sensitivity of ultra-fine grained bulk copper. Although oxidation occurred near the surface, the rate-controlling process was still the deformation of the underlying copper. An increase in the strain rate sensitivity was observed, comparably to that of ultra-fine-grained copper, which can be linked to thermally activated processes at grain boundaries. Important insights into the effects of oxidation on the deformation behavior were obtained by assessing the activation volume. Oxidation of the ultra-fine porous copper foam, thereby hindering dislocations to exit to the surface, resulted in a pronounced reduction of the apparent activation volume from ~800 to ~50 *b* ^3^, as also typical for ultra-fine grained materials.

## Introduction

Nanoporous or ultra-fine porous materials are enormously interesting for a number of future applications due to many excellent properties including: high surface-to-volume ratio, high strength-to-weight ratio, and electrical and thermal conductivity [1]. In addition, it has been shown that materials with high interface fractions can accommodate large amounts of helium and radiation-induced defects, as observed in some nuclear applications [2]. In the past these interfaces were found as Kurdjumov–Sachs interfaces in bcc/fcc metals. However, a free surface can be considered as the ultimate defect sink and, therefore, little damage can accumulate within the metal leading to a rather radiation tolerant material [3]. The excellent properties listed above can be used for combining structural purpose and functional use in the same material. The extraordinary strength-to-weight ratio is based on the fact that decreasing the length-scale of the ligaments to nanometers leads to an increase of the yield strength of the individual ligaments, approaching the theoretical strength of the material [4–7]. Therefore, an individual small scale ligament carries more load than the same volume in a dense bulk material, leading to a weight reduction.

The ligament size and morphology can be controlled during the synthesis process by heat treatments or chemical treatments. Adjusting these parameters will allow tailoring foams for certain purposes.

A classical method to obtain nanoporous structures is selective dissolution from an alloy, which has been used previously to fabricate metal foams such as Ag, Au, Cu, Pd, or Pt [8]. Notably, in this well known approach the first step is the preparation of an alloy consisting of two miscible elements, followed by a dealloying step of one of the elements, resulting in a nanoporous foam with ligaments in the range of few nanometers [8]. We developed another procedure that can avoid the steps of alloying and dealloying. Therefore, in this work, the Cu–Fe system was used, which shows a high immiscibility of Cu and Fe [9] at room temperature. Our approach is to utilize high pressure torsion (HPT) as a powder consolidation step and to subsequently produce the ultra-fine grained (UFG) composite microstructure by applying large amounts of shear deformation. Subsequently, selective dissolution of Fe from the composite results in a remaining ultra-fine porous (UFP) Cu foam structure.

In order to utilize these foams in future applications, it is necessary to acquire information about the foam manufacturing, their thermo-mechanical properties, and the plastic deformation mechanisms even if the application has a functional purpose. Nanoindentation is a well-suited method to obtain mechanical properties for micro- and nanoporous structures with high lateral and depth resolution [4, 5]. Important data can be obtained to determine the dominant deformation processes and mechanical behavior even at elevated temperatures. The rate dependent deformation mechanisms can be obtained locally by strain rate jump, relaxation, or indentation creep tests [10–13]. There are several characteristic properties of a material that can be used for a description of the time and strain rate dependent mechanisms inside the material. The strain rate sensitivity *m* and the activation volume *A* are two of these characteristic properties. Previously, nanoindentation was mostly used to determine *m* and *A* for UFG and coarse-grained (CG) bulk metals [10–21], but not for porous metal foams. In this work, *m* and *A* were determined by local stress relaxation tests in order to obtain more insight into the dominating deformation mechanisms of UFP materials at elevated temperatures.

## Experimental procedures

### Foam manufacturing

The sample material was produced directly from commercially available powders via HPT processing. The powders used were copper (99.9 % purity, −170 + 400 mesh, 37–88 μm) and iron (99.9 % purity, −100 + 200 mesh, 74 – 149 μm) obtained from Alpha Aesa (Ward Hill, USA). Both powders were premixed in a ratio of 50 at.% Cu and 50 at.% Fe (Cu_50_Fe_50_). The mixed powders were consolidated with a novel two-step HPT process, originally introduced by Bachmaier et al. [22]. The resulting product was an 8 mm diameter disk with a thickness of 1 mm. To reduce the amount of forced mechanical mixing between Cu and Fe, a heat treatment was conducted at 500 °C for 1 h in a vacuum furnace (SERIES XRETORT, Xerion Advanced Heating Ofentechnik GmbH, Germany). The vacuum pressure never exceeded 3 × 10^−4^ mbar during the heat treatment. The heating rate of the furnace was 10 °C per minute and then cooling down to RT required 8 h. The solubility of Cu in Fe at 500 °C can be found in [23] between 0.02 and 0.2 wt% and, therefore, it can be stated that in the worst case this amount of Cu will be still in the Fe.

The bulk UFP copper was prepared by selective leaching of iron using a 5 wt% hydrochloric acid (HCl) solution for 35 h at a temperature of 55 °C. After 35 h the samples were removed from the solution and cleaned with acetone and ethanol to remove the residual HCl-solution. This resulted in dissolution of the Fe from the composite top and bottom surface to a depth of ~ 50 µm. Notably, this is much deeper that the subsequent nanoindentation experiments will probe. The compact composite sample core is important, as it serves to prevent penetration of any form of glue or cement used for fixation of the samples for subsequent nanoindentation testing.

### Foam characterization and testing

All microstructural investigations were made in axial and/or tangential direction at a radius of 3 mm on the HPT disks, related to the high grade of deformation at this radius. After the dealloying process the morphologies and structures of the UFP Cu were investigated to confirm a successful dissolution process. To verify the morphologies of the produced UFP Cu throughout, the samples were investigated using scanning electron microscopy. The microstructural investigations were performed in axial direction using a scanning electron microscope (SEM; LEO type 1525, Carl Zeiss GmbH, Germany) equipped with an energy dispersive X-ray spectrometer (EDX) or a dual beam focused ion beam (FIB)-SEM (Quanta 3D FEG, FEI, USA). EDX spectra were collected from the UFP Cu over an axial region to estimate the remaining Fe concentration. A more accurate method to obtain information about the porosity is to evaluate the relative density from micrographs. SEM images were processed using the computer software AnalySIS (AnalySIS Pro 5.0, Olympus Soft Imaging Solutions GmbH, Germany). This phase analysis was performed for SEM images of different magnifications and also compared to manual image analysis in order to identify parameters that deliver repeatable and reliable measurements. Local cross-sectioning of the samples and subsequently SEM imaging was conducted using a Quanta 3D FEG FIB in order to investigate the structure and morphology beneath the surface of the foam.

**Fig. 1:**
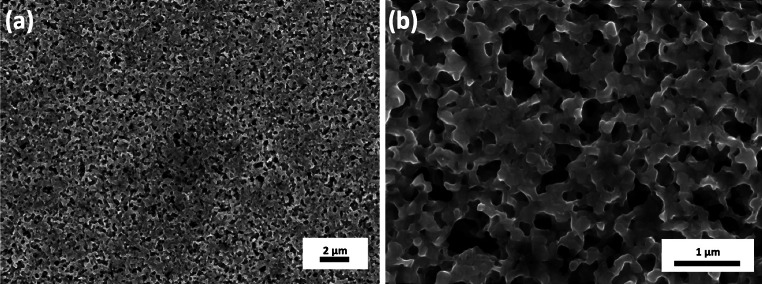
SEM images of the ultra-fine porous Cu showing the obtained foam structure in axial direction after the selective dissolution with a low magnification in **a** and a high magnification in **b**

The mechanical properties of the foam were investigated using a nanoindentation system (Micro Materials NanoTest Platform 3, Micromaterials, UK) with a high temperature option. The machine was placed into an environmental chamber purged with high purity argon to reduce the oxygen level below 2 % aiming to minimize oxidation of the sample. The measurements, data recording, and data evaluation were carried out using the software “NanoTest Platform Three,” Origin, and Microsoft Excel. For RT measurements the samples were fixed with super glue on a common sample holder. For the high temperature measurements the specimens were mounted onto the heater stage with a high temperature cement (Omegabond 600, Omega Engineering Inc., Stamford, USA). The heating system of the indenter consists of resistance heaters at the sample stage and the indenter tip [24]. A thermocouple was mounted onto the sample surfaces of an identical porous reference specimen situated possibly close to the measured sample to control the temperature accurately. A second heating element and thermocouple were used to control the temperature of the indenter tip in order to minimize thermal fluctuations during the indentation process. Additionally, a water-cooled heating shield was inserted to reduce heat load on the heat sensitive parts of the indenter, keeping the heat between the sample and the tip rather than heating axillary component of the system. The entire indentation system is based on a floating table to minimize vibrations. Indentations were performed at RT (22 °C), 50, 100, 200, and 300 °C in argon atmosphere (oxygen content <2 %). For the high temperature measurements a cubic boron nitride (cBN) Berkovich indenter was used up to 300 °C. cBN indenters are preferable over diamond due to the fact that cBN does not disintegrate due to chemical interaction with the cover gas or the specimen [25]. After the high temperature indentation measurements, all samples were measured again with a diamond Berkovich indenter at RT to obtain information about the changes of the mechanical properties. This also allows the comparison of the diamond- and cBN-indenter data. Indentations were performed on the planar, polished surfaces of the samples. The tip calibrations were performed on fused silica before and after each high temperature indentation experiment cycle allowing to calculate the tip area function. All measurements were conducted at a radius of 3 mm from the center of the HPT sample, and the distance between individual indents was at least 50 μm (Fig. 1).

**Fig. 2:**
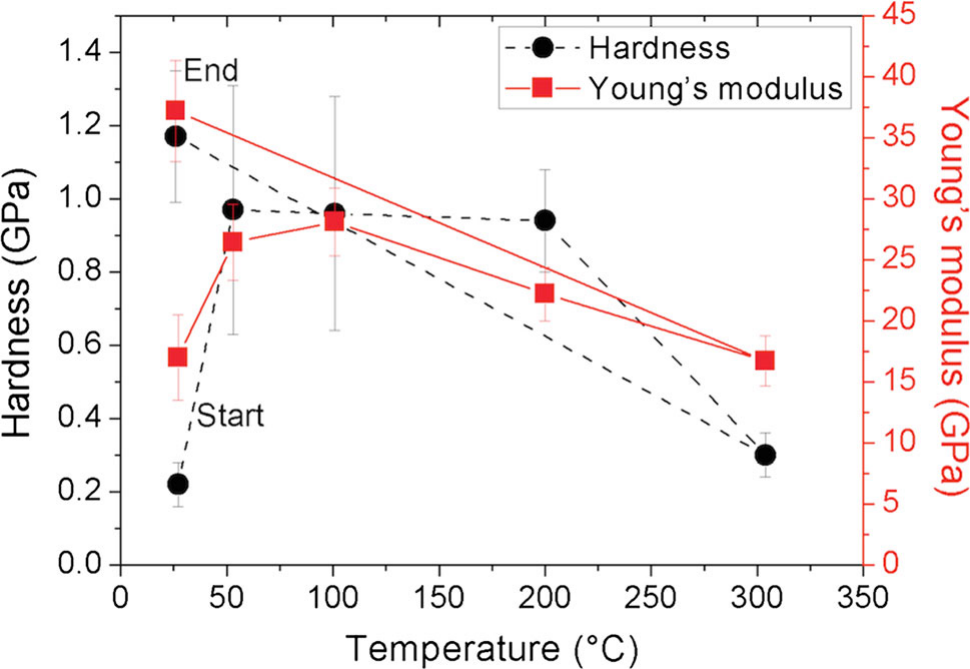
Young’s modulus (*red squares*) and hardness (*black circles*) versus temperature for ultra-fine porous Cu up to 300 °C. The term “End” indicates the RT hardness and Young’s modulus after high temperature experiments (Color figure online)

In order to get reference values for the hardness and Young’s modulus of the UFP Cu, depth controlled (DC) nanoindentations were performed to a total depth of 2000 nm with a constant strain rate of 0.1 s^−1^. The dwell segment for the DC measurements was 30 s and the unloading rate was 10 mN/s. Constant load (CL) relaxation tests were performed to determine the strain rate sensitivity *m* of the material. For the determination of the strain rate sensitivity *m*, it is essential to test similar volumes. Since the material strength increased due to oxidation of the surface (Fig. 2), the maximum loads were adjusted accordingly, ranging from 8 mN for the as-prepared RT measurements to 30 mN for the post-heating testing, in order to always reach the same penetration depth of 2000 nm and thereby keeping the sampled volumes comparable. For all CL measurements a loading time of 10 s, a dwell time of 200 s, and an unloading time of 5 s were used with subsequent drift monitoring of 60 s at 10 % of the peak load. A minimum of 10 indents per temperature and condition were performed. The last 60 % of the recorded drift data was used for the thermal drift correction. The thermal drift of all measurements was below 0.3 nm/s. The evaluation of hardness *H* and the reduced modulus *E*_r_ during this work was performed from the load–displacement curves by the Oliver-Pharr-method [26]. For the sake of simplicity, the near zero Poisson ratio *ν* assumption (*ν* = 0) for low density foams was used for obtaining the Young’s modulus *E* from the reduced modulus and for calculating the flow stress from the hardness of the UFP Cu (*H* = *σ*_f_^*^) [4, 27]. It should be noted here that, while the assumption of a near zero Poisson’s ratio is common, there is an ongoing discussion on the validity of this approach [6, 7], in particular for high density foams such as the one studied here. This will be addressed in more detail later in the manuscript.

The depth-time curve of the dwell period can be separated into two distinct regions, called Stage a and Stage b as proposed by Peykov et al. [11], where Stage a represents the creep behavior within the first 20 s of constant loading and Stage b describes the mechanical behavior up to 200 s.

The relative depth versus time curves of each indentation were fitted with the following empirical function [11]:

1 $$ h_{\text{r}} (t) = A \cdot \left| {h - x_{\text{c}} } \right|^{P} , $$

where *h*_r_ is the relative indentation depth, *h* the actual indentation depth, and *A*, *x*_c_, and *P* are fitting parameters.

The least-square method was used to fit the depth-time curves. The absolute depth *h*_a_ must be used for the further calculation of the displacement rate:

2 $$ h_{\text{a}} \left( t \right) = h_{\text{r}} \left( t \right) + h_{0} . $$

Hereby, *h*_0_ is the indentation depth at the beginning of the dwell period.

The displacement rates $\dot{h}_{\text{a}}$ were derived from the derivative of this fitted curve and subsequently the strain rates $\dot{\varepsilon } = \frac{{\dot{h}_{\text{a}} }}{{h_{\text{a}} }}$ were calculated. The current hardness under load (apparent load divided by area) was obtained from the average load during the hold segment and the projected area (obtained from the original data). Finally, the hardness and strain rate were plotted in a double logarithmic plot to obtain the strain rate sensitivity using linear fits for the two regimes by utilizing the following relations [28, 29]:

3 $$ m = \frac{\partial \ln \sigma }{{\partial \ln \dot{\varepsilon }}} \sim \frac{\partial \ln H}{{\partial \ln \dot{\varepsilon }}}. $$

Hereby, the *m* value for Stage a was assessed from the recorded data of the first 20 s, and the fit of Stage b included data between 30 and 200 s. The data of the transient region between 20 and 30 s was discarded.

The activation volume *A* was obtained for each regime using the following equation [30]:

4 $$ A = \sqrt 3 kT\frac{{\partial \ln \dot{\varepsilon }}}{\partial \sigma } = c^{*} \sqrt 3 kT\frac{{\partial \ln \dot{\varepsilon }}}{\partial H}. $$

Here, *k* = 1.3806488 × 10^−23^ m^2 ^kg s^−2 ^K^−1^ is the Boltzmann constant and c* is the constraint factor, which describes the relation between hardness and flow stress (2.8 for a Berkovich indenter tip at constant 8 % representative strain) [31].

## Results

### Foam manufacturing

The UFP Cu is obtained by selective dissolution. The resulting structure in axial direction is shown in Fig. 1. The ligament diameter is ∼200 nm, as determined from image analysis. While the ligaments at the sample surface could be identified with sufficient precision by digital image analysis, the pores could not be tracked with good accuracy due to the strong contrast variations below the foam surface (see Fig. 1b). Starting from a composite having comparable grain size for Fe and Cu, respectively, one might expect that the pore diameter is of comparable dimension to the ligaments. Moreover, ligament widths and pore sizes are not perfectly homogenous throughout the entire specimen. This is related to the manufacturing process involving shear in only one axis and not in multiple axis. The relative density of this foam structure is 53 ± 1.5 % from analyzing SEM images, and supported by EDX investigations showing ~ 3 % Fe remaining in the foam.

### Young’s modulus and hardness

To access the change of hardness *H* and Young’s modulus *E* over temperature, DC measurements were performed with a constant strain rate. The data points marked as red squares in Fig. 2 visualize the correlation between Young’s modulus, which was obtained from the reduced modulus, and temperature. The values for 22 °C in the non-oxidized state are indicated by the label “Start.” First, an increase of the Young’s modulus from 17 ± 3.5 GPa to 26.8 ± 3 GPa was observed up to 100 °C, which was followed by a decrease in modulus down to 22.2 ± 2.2 GPa at 200 °C and 16.7 ± 2.1 GPa at 300 °C. The data points marked as black circles in Fig. 2 show the change of hardness over temperature. A strong increase of the hardness from 220 ± 60 MPa at 22 °C up to ~ 950 ± 300 MPa at 50 °C was observed, which stayed constant at 100 and 200 °C. At 300 °C, a hardness drop from 940 ± 140 MPa to 300 ± 60 MPa was observed. The values of the RT hardness after the high temperature experiments at 22 °C are in both graphs close indicated as “End.” The sample after high temperature indentation shows an enormous increase of around 500 % in hardness and 200 % in Young’s modulus compared to the original 22 °C experiments.

### Strain rate sensitivity

**Fig. 3:**
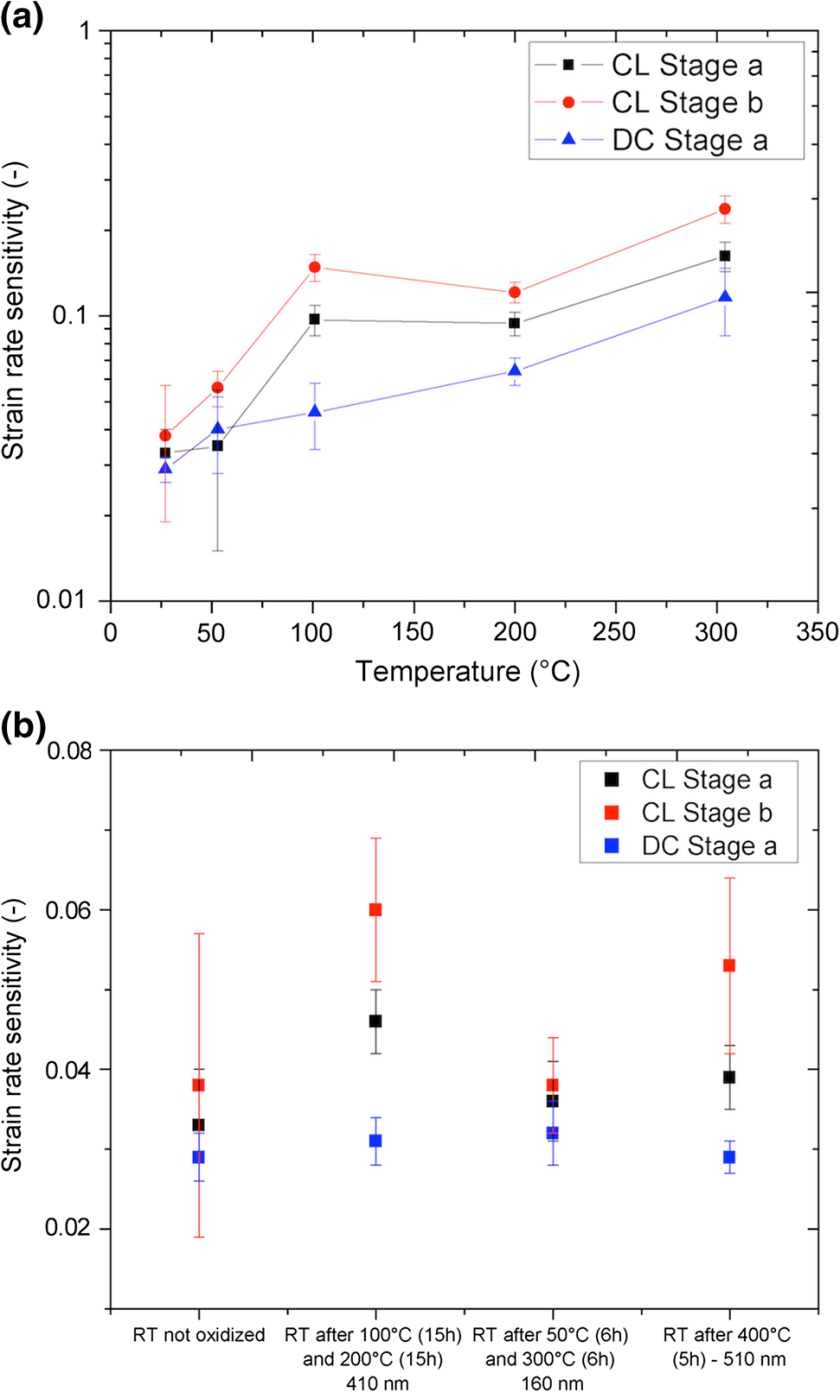
**a** Strain rate sensitivity over temperature for CL and DC measurements. **b** Strain rate sensitivity for a non-oxidized and differently oxidized UFP Cu samples at RT. The tick labels on the *x*-axis give the duration of the heating segment and the estimated thickness of the oxide layer

Figure 3a shows the *m* value versus temperature for CL and DC measurements. In general, an increase of the strain rate sensitivity of almost a magnitude from 0.03 to 0.1–0.2 between RT and 300 °C was observed for all different experimental conditions. For Stage a and Stage b of the CL experiments the same trend of the *m* value was observed. The results of the RT experiments after high temperature indentation are shown in Fig. 3b. No significant changes of the *m* value between the measurements before and after oxidation were observed for the DC measurements, which show a rather constant value of 0.03 for all different conditions. The Stage a *m* values of the LC measurements are approximately 0.04 and show higher scattering than the DC measurements. The Stage b results of LC measurements are between 0.04 and 0.06 and exhibit significantly higher standard deviations.

### Activation volume

**Fig. 4:**
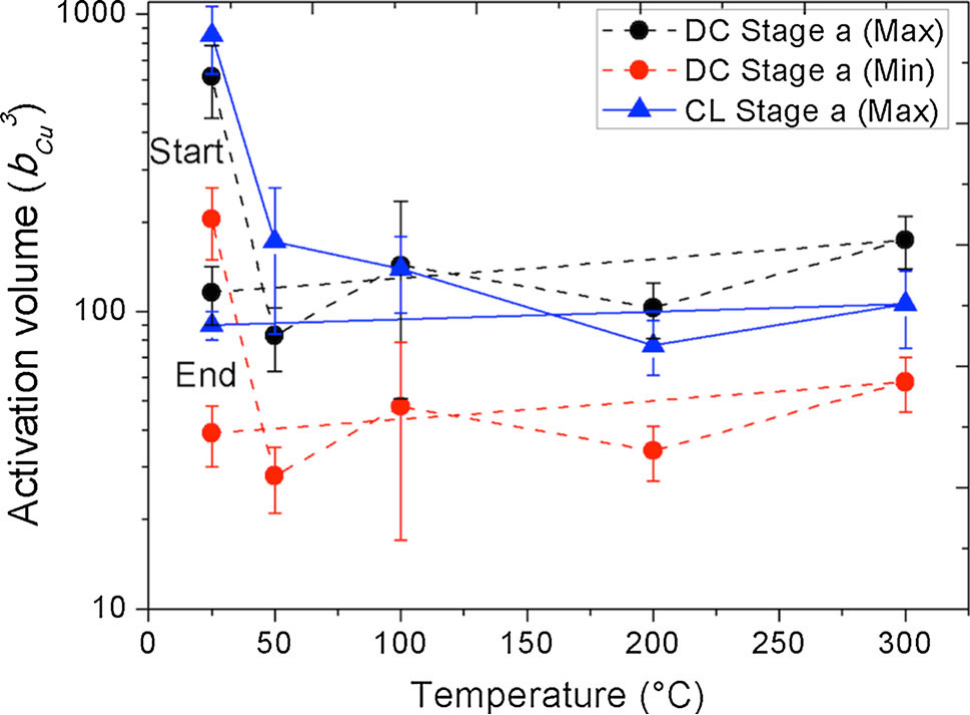
Activation volume *A* obtained from DC to CL measurements. The minimal and maximal normalized *A* for DC measurements are plotted. For comparison maximal CL Stage a results are also shown. A detailed explanation is given in the text

The activation volume versus temperature is plotted in Fig. 4. The activation volume is given in units of the cubic Burgers vector for Cu (Dislocation: 1/2 {110}, *b* = 0.255 nm [32]). As mentioned previously, there is an ongoing debate on the actual Poisson’s ratio for porous materials, in particular for high density foams, whether it is near zero [4, 27] or approaching the bulk value [6, 7]. Thus, to account for this controversy, an upper and lower boundary calculation of the activation volume using Eq. (4) was performed. The upper boundary is given by *H* ~ 3 *σ*_f_ (behavior of high density foams), and the lower boundary by *H* ~ *σ*_f_. These boundaries are also regarded to encompass effects emerging from the densification of the foam during oxidizing and the correlation for ceramics flow stress and hardness of *H* ~ 1.5 *σ*_f_. The minimal (red data) and maximal (black data) activation volume versus temperature for DC measurements are plotted in Fig. 4. The activation volume strongly decreases from approximately 250–850 *b* ^*3*^ at RT to 20–150 *b* ^*3*^ at 50, 100, 200, and 300 °C, respectively. Thus, *A* remains rather constant at elevated temperatures. The activation volume for the oxidized sample is about 10–15 times lower (10–100 *b* ^*3*^) than that of the non-oxidized material. Generally, the same trend and activation volumes as for CL-tests (Stage a) were observed as shown for comparison in Fig. 4 (blue data) for the upper boundary values. Here it is to mention that CL Stage b values are not shown in this figure, but Stage a and Stage b values show a nearly identical trend.

## Discussion

### Mechanical properties

**Fig. 5:**
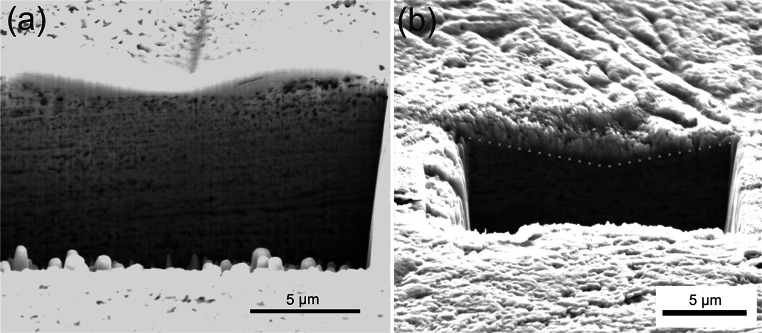
**a** SEM image of a local cross-section beneath a residual impression in the non-oxidized state. **b** Local cross-section showing a rather dense near surface oxide layer on top of the UFP Cu after 100 °C/6 h and 200 °C/6 h. The *blue line* indicates the oxide-bulk interface (Color figure online)

The hardness values of CuO and Cu_2_O are between 2050–2490 and 2010–2030 MPa [33], respectively, while the yield strength value of compact UFG Cu with a grain size around 200 nm is ~450 MPa [34, 35]. This results in an estimated hardness for UFG Cu of 1350 MPa (*H* = 3 *σ*_f_). Thus, the difference in hardness between copper and copper oxide is significant. The increase of Young’s modulus cannot only be explained by the values of CuO and Cu_2_O, which are 80 and 30 GPa [36, 37], respectively, because during the oxidation of Cu to copper oxide (mainly Cu_2_O), the relative density of the foam increases due to an increase in volume of the oxide. This is because of the uptake of oxygen from the atmosphere. The volume of the foam would increase by 40–45 % if the copper completely converts to copper oxide. Thus, the relative density would be ~95 %. In Fig. 5a a local cross-section of a residual impression in the non-oxidized state and in Fig. 5b in the oxidized state after high temperature experiments for 6 h at 100 and 200 °C, respectively, are shown. The increase of relative density up to 95 % close to the surface can be clearly seen when comparing Fig. 5a, b. Furthermore, the roughness has increased dramatically, resulting in a relatively high standard deviation of hardness and Young’s modulus values (see Fig. 2). This is related to an inhomogeneous oxidation process of the surface (see Fig. 5b) and to an increased drift influence at elevated temperatures. The local cross-section also shows that the first 1–2 μm of the surface are completely oxidized and seemingly dense, even after low temperature oxidation of the UFP Cu at 100 °C for 6 h and 200 °C for 6 h. The zone below the oxidized layer, however, is not visually affected by the oxidation. Another possible explanation of deviating values is that there is a hard oxide layer on the soft UFP Cu, which can cause a sink-in and therefore, an underestimation of the measured values. But this cannot be proved due to an ongoing oxidation process during the measurements.

The flow stress of the foam can be compared to values predicted by scaling laws. The flow stress of the UFP Cu was assessed from the indentation experiments (for low density foams: *H* = *σ*_f_^*^ [27]). According to this, the flow stress of the UFP Cu investigated in the present study is 220 MPa at 22 °C. The yield strength for bulk copper with an average grain size of 200 nm is 450 MPa [34, 35]. Classically, the yield strength *σ*_ys_^*^ of the foam can be obtained from the scaling law [27]:

5 $$ \sigma_{\text{ys}}^{*} = C_{ 1} \cdot \sigma_{\text{ys}} \cdot \left( {\rho^{ *} /\rho_{\text{s}} } \right)^{n}, $$

where *σ*_ys_, *ρ*_s_, and *ρ* ^*^are the yield strength of the cell wall material, the density of the solid, and the density of the foam, respectively (*C*_1_ = 0.3 and *n* = 1.5 [27]). For the present case, the flow stress and the yield strength of the foam are compared for a good estimation. The experimentally determined value of flow stress is nearly five times larger than the value predicted by Eq. (5), 220 MPa instead of 52 MPa. This deviation gives rise to the question whether the scaling laws deduced from macroscopic low density foams [27] can still be applied to nanoporous or UFP materials [7]. In addition, further possible explanations for this difference are:

- The existence of additional strengthening from supersaturated Fe in the Cu ligaments [23].
- The Gibson and Ashby equation is predominantly valid for homogenous foam structures and lower relative densities [27].
- The assumed near zero Poisson’s ratio is not fully valid for relative densities >35 % such as in the present UFP Cu [27].
- The used yield strength for bulk Cu with ~ 200 nm grain size is just an estimation, the structure size in the UFP Cu varies from ~ 100–300 nm.
- Even a thin oxide layer can cause a pileup of dislocations and, therefore, strongly influence the material properties.

For a further discussion, we assume that the Ashby and Gibson Eq. (5) describes the mechanical properties of the UFP Cu well [27]. Then, the experimentally determined value of 220 MPa for the yield strength of the UFP Cu would require that the yield strength of the foam ligaments is in the order of 1.9 GPa. This interpretation suggests that the yield strength of the ligaments in the UFP Cu approaches the theoretical strength of Cu (>6 GPa [38]), as it was already achieved for nanoporous Au foams by Biener et al. [5].

The Young’s modulus of the UFP Cu was determined as 17 GPa at 22 °C. Assuming that the UFP Cu sample exhibits a relative density of 53 % and using the following equation from Ashby and Gibson [27]:

6 $$ E^{ *} = C_{2} \cdot E_{\text{s}} \cdot (\rho^{ *} /\rho_{\text{s}} )^{n} , $$

the scaling law would predict a Young’s modulus of 28–36 GPa. Whereby in Eq. (6), *E*_*s*_ (for Cu *E* = 100–130 GPa), *ρ*_s_, and *ρ* ^*^ are the Young’s modulus, the density of the solid material, and the density of the foam, respectively (*C*_2_ = 1 and *n* = 2 [27]). The difference of 10–20 GPa between model and experiment can be explained by the layered-structure of the UFP Cu from the HPT shear deformation process, which is shown in Fig. 5a, b. The ligaments in the direction of indentation do not exist in the same density as the ligaments perpendicular to them, which is related to the shear deformation process. The outcome of this is a lower Young’s modulus in loading direction.

**Fig. 6:**
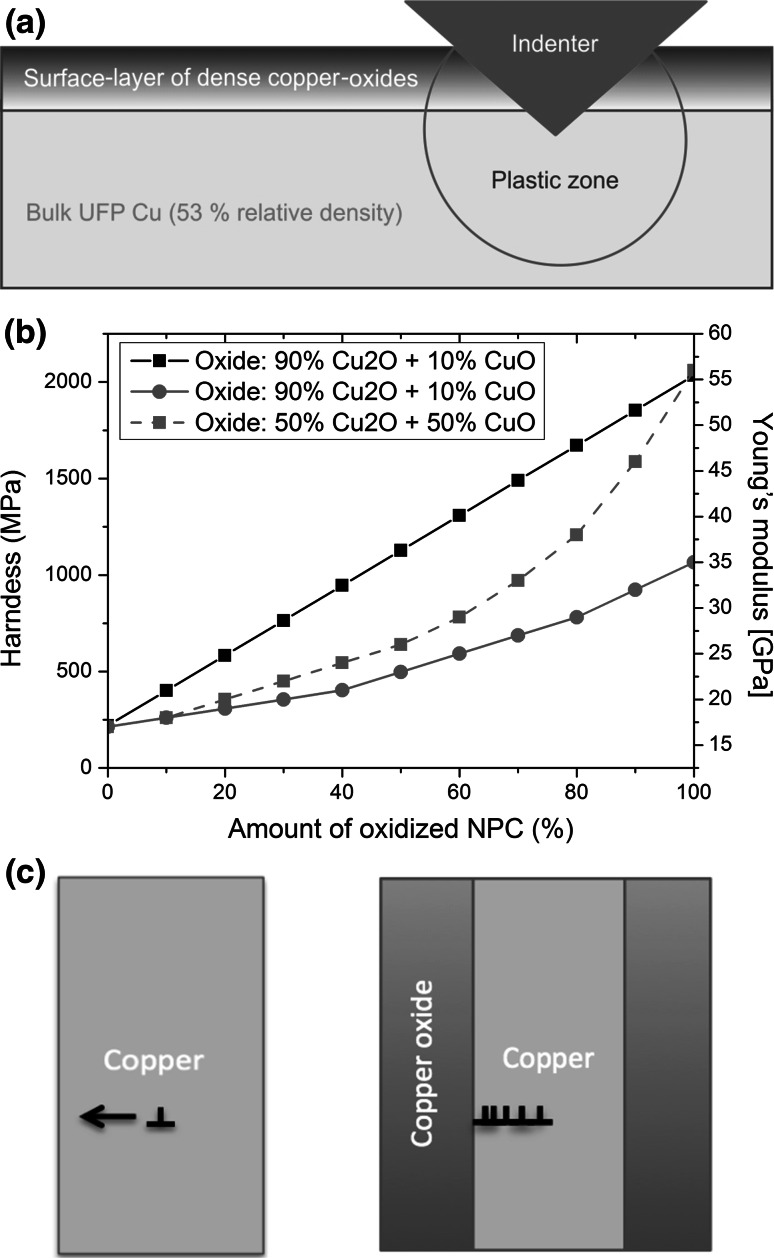
**a** Schematic model of the oxidation behavior of the ultra-fine porous Cu influencing the plastic zone under the indenter tip. **b** Hardness and Young’s modulus for different relative amounts of oxidized UFP Cu in the plastic zone. **c** Schematic of a non-oxidized Cu ligament, where dislocations can easily exit to the sample surface, and a partially oxidized ligament, where dislocations are trapped inside the Cu phase of the ligament and pileup at the oxide

In order to understand the changes of hardness and Young’s modulus, a model is proposed to predict the hardness for different oxidized samples at 22 °C. During oxidation of the UFP Cu, an increase in volume of ~ 45 % occurred. Therefore, the assumption of a dense oxide layer, which is growing from the top of the UFP Cu, can be used for developing a model as shown in Fig. 6a. This composite model will allow a prediction of the hardness and stiffness characteristics of the oxidized UFP Cu. The following expression describes the ratio of oxide to foam in the area of the plastic zone and allows estimating the hardness:

7 $$ H_{\text{C}} = H^{ *} \cdot v^{ *} + H_{\text{O}} \cdot v_{\text{O}} .$$

Here, *H*_c_ is the hardness of the composite, *H* ^*^ the hardness of the foam, *ν* ^*^ the fraction of the foam, and *H*_0_ and *ν*_*0*_ are the corresponding values for the oxide. A similar approach was previously suggested by Hosemann et al. to account for the presence of a hardened surface layer due to irradiation [39]. For the approximation of the modulus of the composite, the following equation from the composite theory is used [40]:

8 $$ E_{\text{C}} = \frac{{E^{ *} \cdot E_{\text{O}} }}{{E_{\text{O}} v^{ *} + E^{ *} v_{\text{O}} }}. $$

Here, *E*_C_ is the Young’s modulus of the composite, *E* ^*^of the UFP Cu and *E*_0_ of the oxide, while *ν* ^*^ and *ν*_0_ are the particular fractions of the UFP Cu and oxide. This equation is derived from the composite theory for the Young’s modulus according to fiber-reinforced composites, whereby the load direction is perpendicular to the fiber direction [41].

This allows an estimation of the hardness values, but for estimating the Young’s modulus a detailed knowledge of the oxide composition after different oxidation times and temperatures is necessary. Literature reviews exist regarding the oxidation temperature and time, and the resulting ratios of different copper oxides [42–46]. Not only the thickness of the grown oxide is crucial, but also the type of oxide after different oxidation temperatures plays a major role.

Figure 6b shows the results of the model for obtaining hardness and Young’s modulus values depending on the relative amount of oxide in the plastic zone. The prediction for the hardness is quite accurate due to the nearly identical hardness of the oxides. A precise estimation of the Young’s modulus is not possible, since the ratio of different oxides is unknown. A change in the ratio of the oxides between 10 and 50 % as used here was also reported by Lenglet et al. [47].

### Strain rate sensitivity

**Fig. 7:**
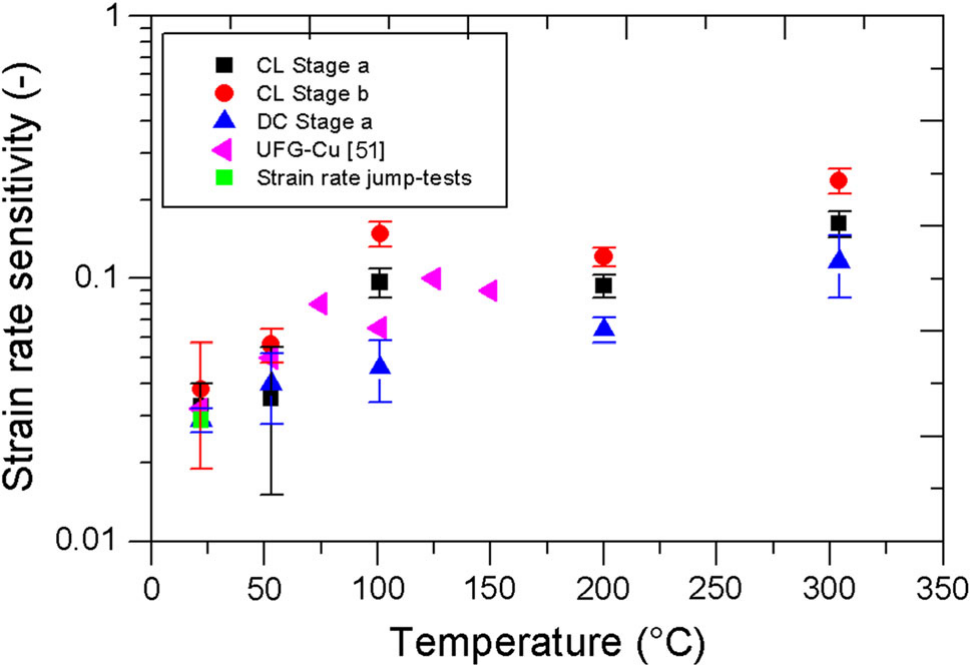
Strain rate sensitivity over temperature for the ultra-fine porous Cu (*black squares*, *red circles*, *blue triangles*, *green squares*) and for bulk UFG Cu (*pink triangles*) (Color figure online)

The examination of the strain rate sensitivity *m* and the activation volume *A* is necessary in order to understand the deformation behavior of the material. All obtained *m* values from DC and CL measurements for Stage a and Stage b at RT are around 0.03–0.04 in the non-oxidized state (Fig. 7). Thus, *m* is similar to that of bulk UFP Cu with a grain size around 150–200 nm (0.03 as summarized by Chen et al. [21]), as shown in Fig. 7. Besides the stress relaxation tests, strain rate jump tests were performed on the UFP Cu at RT in order to double check the obtained results [13]. The strain rate jumps were performed at an indentation depth of 1000 and 1500 nm from 0.05 to 0.001 s^−1^ (Fig. 7). These tests revealed a similar strain rate sensitivity of 0.026–0.029 for the NPC. The high value of *m*, compared to CG materials, is related to the increased amount of grain boundaries, which influence the dislocation mobility of the material [35]. Therefore, the behavior of the UFP Cu and bulk UFG Cu concerning the strain rate sensitivity are similar, even at elevated temperatures. After oxidation of the UFP Cu, *m* is still in the same order at RT (~ 0.03–0.04) for Stage *a* tests (Fig. 3b). Thus, during stress relaxation tests the rate-controlling phase has to be the copper and not the copper oxides. This is because of a quasi-elastic behavior of the rather dense oxide on top of the foam and a plastic behavior of the underlying copper ligaments. Copper oxide (Cu_2_O) polycrystals are reported to have a very limited dislocation mobility [37, 48], thus resulting in a brittle behavior up to 300 °C.

The increasing strain rate sensitivity over temperature from 0.03 to 0.1–0.2, shown in Fig. 7, can be explained by thermally activated climb-controlled annihilation of lattice dislocations in the Cu ligaments, which favorably takes place at high angle grain boundaries [49, 50]. The large fraction of high angle grain boundaries is a consequence of the HPT process. The high misorientation and amount of high angle grain boundaries enhances climbing controlled processes, even at low temperatures. At elevated temperature (100–300 °C) a similar behavior was found for equal channel angular pressing (ECAP) UFG Al by Vevecka-Priftaj et al. [50] and for ECAP UFG Cu by Bach et al. [51] (Fig. 7).

### Activation volume

The obtained activation volume for the UFP Cu at 22 °C does not correlate with the values of bulk UFG Cu with the same structure size. The activation volume for non-oxidized UFP Cu is in the order of 250–850 *b* ^3^ when taking the upper and lower boundary for the hardness to yield strength conversion into account. This activation volume indicates a dominant deformation behavior governed by interaction between lattice and forest dislocations, which is typical for CG Cu (~ 1000 *b* ^3^). Lower values of ~ 100 *b* ^3^ were observed for UFG Cu [18] and UFG Al [14]. The obtained activation volume for the oxidized sample is within the same order of magnitude, namely 50–150 *b* ^3^ (Fig. 4). Hereby, giving the activation volume in units of *b* ^3^ of Cu is feasible due to the nearly same Burgers vector for the 1/2·(110) dislocation in Cu (*b* = 0.255 nm [32]) and the 1/2·(100) dislocation in Cu_2_O (*b* = 0.213 nm [52]). In general, after oxidation the density of the UFP Cu increases and an oxide layer grows, while the measured activation volume significantly drops from 250–850 to 20–100 *b* ^3^ (Fig. 4). Polycrystalline copper oxides do not extensively plastically deform in a temperature range from 22 °C up to 300 °C at atmospheric pressure [52, 53]. Thus, the reason for the drop in activation volume is that the oxide layer on top of the Cu ligaments traps dislocations inside the Cu ligaments and thereby strongly reduces the activation volume of the foam, as schematically shown in Fig. 6c. Dislocations are not able to glide from the Cu phase into the ceramic phase due to differences of the crystal structure, and the free path for dislocations to move inside the plastic zone is thus reduced. Additionally, stress is induced close to the Cu–CuO interface in the copper phase by the effect of epitaxial strains due to relatively high difference in the Pilling–Bedworth ratio (oxide–metal volume ratio), which hinders dislocation movement close to the interface. Contrarily in the case of the non-oxidized Cu ligaments shown in Fig. 6c, the dislocation mobility is not influenced by surface oxides and the dislocations can exit to the surface. Both, the CL and DC measurements show a sudden decrease of *A* immediately after first oxidation occurred (Fig. 4). After this first drop the activation volume up to 300 °C approximately remains on the same level of 50–150 *b* ^3^, comparable to UFG bulk Cu where grain boundaries hinder the dislocation movement.

## Summary

In conclusion, nanoindentation experiments between room temperature and 300 °C were successfully conducted on UFP Cu. During testing at elevated temperatures, an oxidation of the copper occurred. Increasing hardness and Young’s modulus were observed with increasing indentation temperature, which is related to the oxidation of the copper foam. A model was developed taking into account the mechanical properties and growing rates of the copper oxides, which allows an explanation of the measured mechanical properties in dependence on the proceeding oxidation. The oxidation did not significantly affect the rate dependent properties of the UFP Cu since the oxide mostly deforms elastically. The strain rate sensitivity of 0.03 at RT is in the range of UFG bulk copper [21, 35]. Furthermore, an increase of the strain rate sensitivity from 0.03 at RT to 0.1–0.2 at 300 °C was observed, which can be linked to more pronounced thermally activated grain boundary processes at elevated temperatures [50, 51]. The activation volume was strongly influenced by the oxidation due to a change in deformation mechanism. Hereby, the oxide layer on top of the ligaments hindered dislocations to exit to the surface and dislocations were piled-up at the oxide-metal interface.
